# Two new species of *Fuscoporia* (Hymenochaetales, Basidiomycota) from southern China based on morphological characters and molecular evidence

**DOI:** 10.3897/mycokeys.61.46799

**Published:** 2019-12-12

**Authors:** Qian Chen, Yu-Cheng Dai

**Affiliations:** 1 Beijing Advanced Innovation Center for Tree Breeding by Molecular Design, Beijing Forestry University, Beijing 100083, China Beijing Forestry University Beijing China; 2 Institute of Microbiology, Beijing Forestry University, Beijing 100083, China Beijing Forestry University Beijing China

**Keywords:** Hymenochaetaceae, phylogeny, taxonomy, wood-rotting fungi

## Abstract

*Fuscoporia* (Hymenochaetaceae) is characterized by annual to perennial, resupinate to pileate basidiocarps, a dimitic hyphal system, presence of hymenial setae, and hyaline, thin-walled, smooth basidiospores. Phylogenetic analyses based on the nLSU and a combined ITS, nLSU and RPB2 datasets of 18 species of *Fuscoporia* revealed two new lineages that are equated to two new species; *Fuscoporia ramulicola***sp. nov.** grouped together with *F. ferrea*, *F. punctatiformis*, *F. subferrea* and *F. yunnanensis* with a strong support; *Fuscoporia acutimarginata***sp. nov.** formed a strongly supported lineage distinct from other species. The individual morphological characters of the new species and their related species are discussed. A key to Chinese species of *Fuscoporia* is provided.

## Introduction

*Fuscoporia*Murrill (1907) was established based on *F. ferruginosa* (Schrad.) Murrill. However, the genus has been unconsidered for a long time, reduced as a synonym of *Phellinus* Quél. (e.g., Overholts 1953; Ryvarden and Johansen 1980; Larsen and Cobb-Poulle 1990). Fiasson and Niemelä (1984) firstly used morphological features to segregate some members of *Phellinus* into distinct taxonomic entities, including *Fomitiporia* Murrill, *Fulvifomes* Murrill, *Phellinus* Murrill, *Porodaedalea* Murrill and *Fuscoporia*.

Fiasson and Niemelä (1984) defined *Fuscoporia* by annual to perennial, resupinate to pileate basidiocarps, a dimitic hyphal system with generative hyphae in the dissepiment edge and the tube trama often encrusted with crystals, presence of hymenial setae and hyaline, thin-walled, smooth basidiospores. Later on, phylogenetic studies based on nuclear large subunit (nLSU) ribosomal RNA-based phylogeny confirmed that *Fuscoporia* formed a lineage distinct from *Phellinus* s. s. (Wagner and Fischer 2001, 2002). Previous studies on *Fuscoporia* were mostly based on morphological characteristics (Groposo et al. 2007; Baltazar et al. 2009; Baltazar and Gibertoni 2010; Raymundo et al. 2013) but, recently, more new taxa were described based on both molecular analyses and morphology (Niemelä et al. 2001; Jang et al. 2012; Pires et al. 2015; Chen and Yuan 2017; Chen et al. 2019).

In our study, phylogenetic analyses were carried out based on the nLSU and combined ITS, nLSU and RPB2 datasets including 99 (60 newly generated) sequences representing 18 species of *Fuscoporia*. From the analyses, two new species of *Fuscoporia* were found and described. In addition, a key to Chinese species in the genus was provided.

## Materials and methods

### Morphological studies

The studied specimens are deposited in the herbarium of the Institute of Microbiology, Beijing Forestry University (BJFC). Macro-morphological descriptions are based on field observations and notes and dry herbarium specimens. The microscopic analyses followed that described by Cui et al. (2019). Sections were studied at ultimate magnification ×1000 applying Nikon Eclipse 80i microscopy and phase contrast illumination. Drawings were made with the aid of a drawing tube. The measurements and drawings were made from slide preparations stained with Cotton Blue. In recording spore size variation, 5% of measurements were excluded from each end of the range and given in parentheses. The following abbreviations are used in the article: KOH = 5% potassium hydroxide, CB = Cotton Blue, CB- = acyanophilous, IKI = Melzer’s reagent, IKI- = neither amyloid nor dextrinoid, L = mean spore length (arithmetic average of all spores), W = mean spore width (arithmetic average of all spores), Q = variation in the L/W ratios between specimens studied, and n (a/b) = number of spores (a) measured from given number of specimens (b). Special color terms are cited from Petersen (1996).

### DNA extraction and sequencing

Extract total genomic DNA was extracted from dried specimens by CTAB rapid plant genome extraction kit (Aidlab Biotechnologies Co., Ltd., Beijing, China) according to the manufacturer’s instructions with some modifications (Chen et al. 2016; Han et al. 2016). To generate PCR amplicons, the following primer pairs were used: ITS4 and ITS5 (White et al. 1990) for the internal transcribed spacer (ITS), LR0R and LR7 (Vilgalys and Hester 1990) for nuclear large submit (nLSU) and bRPB2-6F and bRPB2-7.1R (Matheny 2005) for partial RNA polymerase II, second largest submit (RPB2). The PCR procedures followed Song and Cui (2017) and Zhu et al. (2019). DNA sequencing was performed at Beijing Genomics Institute and the sequences are deposited in GenBank and listed in Table 1.

**Table 1.** Taxa and GenBank accession numbers for ITS, nLSU and RPB2 sequences used in the phylogenetic analyses (Fig. 2).

Species	Sample no.	Locality	GenBank accession no.		
			ITS	nLSU	RPB2
*Fuscoporia acutimarginata*	Dai 15137	China	**MH050751**	**MH050765**	**MN159384**
	Dai 16892	China	**MH050752**	**MH050766**	**MH079393**
	Dai 15115	China	**MN121764**	**MN121823**	**MN159385**
*F. atlantica*	SP 445618	Brazil	KP058515	KP058517	–
	SP 465829	Brazil	KP058514	KP058516	–
*F. callimorpha*	Dai 17388	Brazil	**MN121765**	**MN121824**	–
	Dai 17389	Brazil	**MN121766**	**MN121825**	–
*F. contigua*	Dai 16025	USA	MG008401	MG008454	**MH079406**
	JV 1204/22.6-J	USA	MG008403	MG008456	**MH079407**
	Dai 13567A	Romania	**MG008402**	**MG008455**	**MN159386**
*F. ferrea*	MUCL 45984	France	KX961112	KY189112	–
	Cui 11801	China	KX961101	KY189101	**MN159387**
	JV 1105/3-J	USA	**MH050760**	**MH050770**	**MH079392**
*F. ferruginosa*	JV 1309/4	Slovakia	KX961102	KY189102	**MH079405**
	JV 1507/11-CN	Europe	MG008400	MG008453	**MH079404**
*F. gilva*	Cui 11209	China	**MN121767**	**MN121826**	**MN159388**
	Dai 15681	China	**MN121768**	**MN121827**	**MN159389**
*F. insolita*	SP 5251	Russia	KJ677113	–	–
	SP 5208	Russia	**MN121769**	**MN121828**	–
*F. punctatiformis*	Doll#872	USA	**MH050753**	–	–
	Dai 17443	Brazil	**MH050755**	**MH050764**	–
*F. ramulicola*	Dai 15723	China	**MH050749**	**MH050762**	**MH079398**
	Dai 16155	China	**MH050750**	**MH050763**	**MH079399**
*F. rufitincta*	JV 1008/25	USA	KJ940029	KX058575	–
	JV 0904/142	USA	KJ940030	KX058574	–
*F. senex*	KUC 20110922-13	Korea	JX463658	JX463652	–
	MEL:2382630	Australia	KP012992	KP012992	–
*F. setifer*	Dai 15710	China	**MH050758**	**MH050767**	**MN159390**
	Dai 15706	China	**MH050759**	**MH050769**	**MN159391**
*F. subferrea*	Dai 16326	China	KX961097	KY053472	**MH079400**
	Dai 16327	China	KX961098	KY053473	**MH079401**
*F. torulosa*	JV 1405/2	Czech	KX961106	KY189106	**MN159392**
	JV 1312/19-Kout	Spain	KX961107	KY189107	**MN159393**
*F. viticola*	JV 0911/6	Czech	KX961110	KY189110	–
	He 2081	USA	**MN121770**	**MN121829**	–
*F. wahlbergii*	Dai 15636	China	MG008397	MG008450	**MH079402**
	Dai 15659	China	MG008398	MG008451	**MH079403**
*F. yunnanensis*	Cui 8182	China	**MH050756**	–	**MN159394**
	Dai 15637	China	**MH050757**	**MH050768**	**MN159395**
*Coniferiporia sulphurascens*	Cui 10429	China	KR350565	KR350555	–
*C. weirii*	CFS 504	Canada	AY829341	AY829345	–

Note: New sequences produced by this work are in bold.

### Phylogenetic analyses

**Figure 1.**
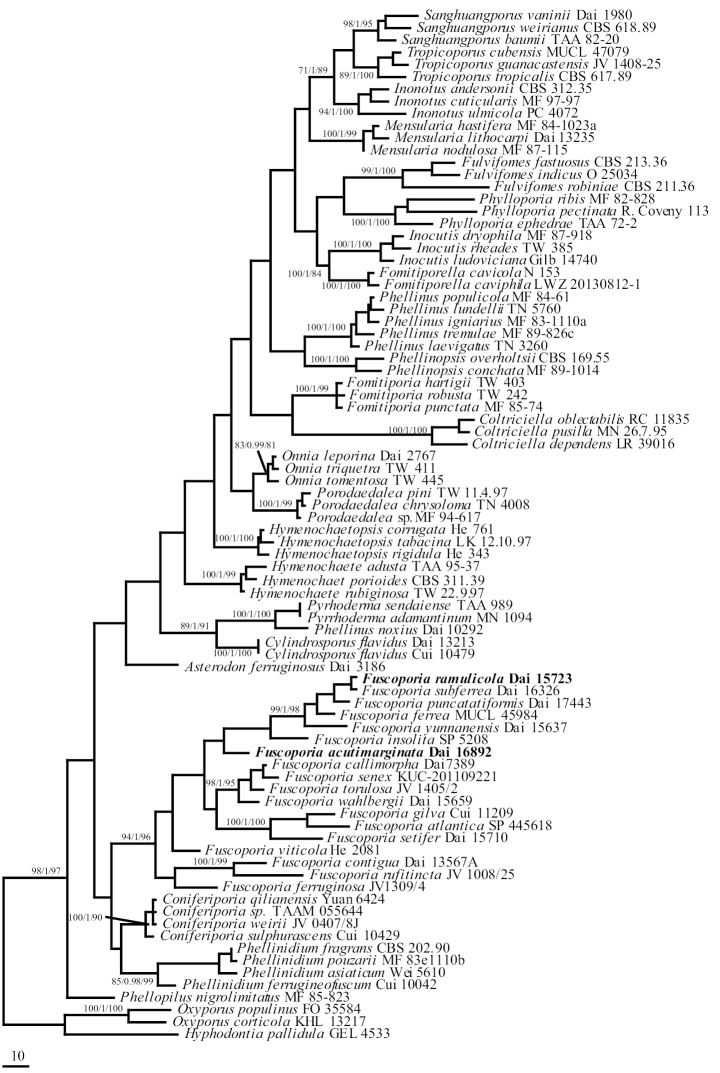
Phylogenetic positions of *Fuscoporia* and the new species within the Hymenochaetaceae inferred from the nLSU sequences. Topology is from MP tree and statistical values (MP/BI/ML) are indicated for each node that simultaneously received BS from ML and MP not below 75%, and BPP from BI not below 0.9. Names of new species are in bold.

**Figure 2.**
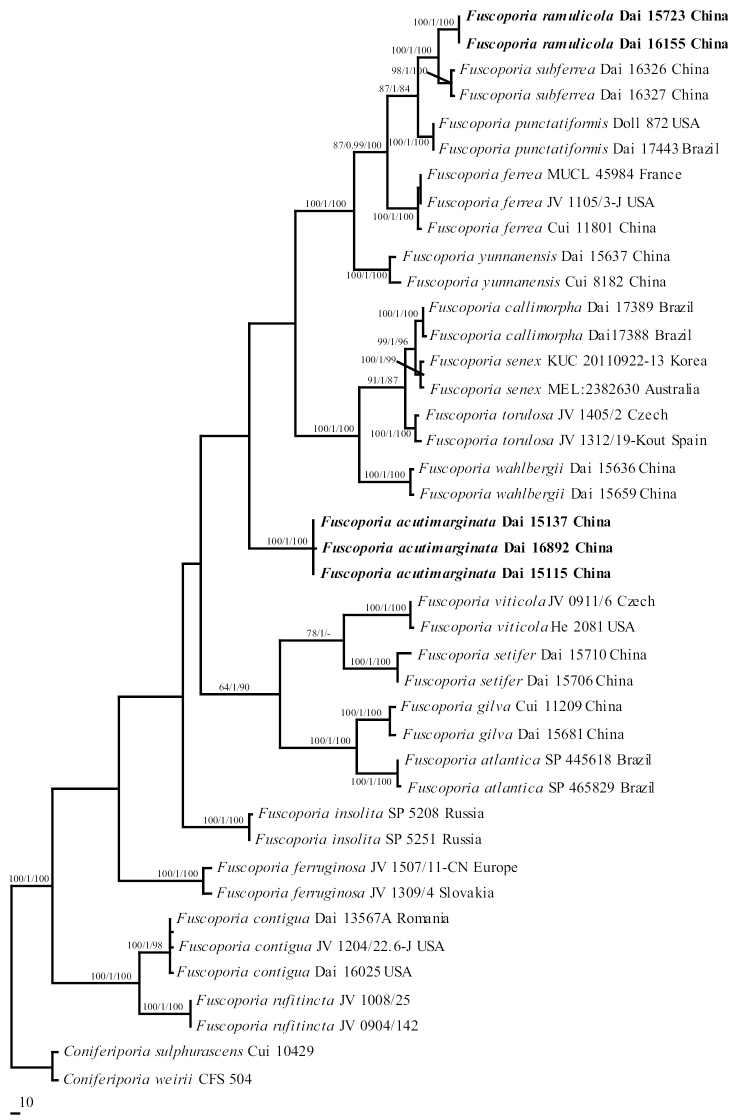
Phylogeny of *Fuscoporia* inferred from ITS+nLSU+RPB2 sequences. Topology is from MP tree and statistical values (MP/BI/ML) are indicated for each node that simultaneously received BS from ML and MP not below 75%, and BPP from BI not below 0.9. Names of new species are in bold.

Sixty new sequences (nineteen ITS, seventeen nLSU and twenty-four RPB2) of *Fuscoporia* species were newly generated (Table 1). All sequences of ITS+nLSU+RPB2 analysis (Fig. 2) were shown in Table 1. Additional sequences of representatives genera of Hymenochaetaceae included in nLSU analysis (Fig. 1) were downloaded from GenBank to explore the phylogenetic relationships of *Fuscoporia*, which were used in the previous phylogenetic study (Zhou et al. 2016; Chen et al. 2019), thus not shown in Table 1. *Oxyporus corticola* (Fr.) Ryvarden, *Oxyporus populinus* (Schumach.) Donk, and *Hyphodontia pallidula* (Bres.) J. Erikss. were included as outgroups in nLSU analysis based on previous studies (Zhou et al. 2016; Chen et al. 2019). The outgroups selected for ITS+nLSU+RPB2 analysis were *Coniferiporia weirii* (Murrill) L.W. Zhou & Y.C. Dai and *Coniferiporia sulphurascens* (Pilát) L.W. Zhou & Y.C. Dai because *Coniferiporia* resulted as a sister group of *Fuscoporia* in previous studies (Fig. 1; Zhou et al. 2016; Chen and Yuan 2017; Chen et al. 2019).

Sequences were aligned with BioEdit (Hall 1999) and ClustalX (Thompson et al. 1997). Prior to phylogenetic analysis, ambiguous sequences at the start and the end were deleted and gaps were manually adjusted to optimize the alignment. Sequence alignment was deposited at TreeBase (http://purl.org/phylo/treebase; submission ID 22620). Phylogenetic analysis was carried out according to previous studies (Zhou 2015; Shen et al. 2019; Zhu et al. 2019). Maximum parsimony (MP), bayesian inference (BI) and maximum likelihood (ML) methods were employed to perform phylogenetic analysis of the two aligned datasets. MP analysis were performed using PAUP* 4.0b10 (Swofford 2002); BI was calculated with MrBayes3.1.2 (Ronquist and Huelsenbeck 2003); RAxML v.7.2.6 (Stamatakis 2006) was used for ML analysis. The three phylogenetic methods resulted in similar topologies for each dataset, and, thus, only the topology from the MP tree is presented along with statistical values from the ML/BI/MP algorithms (simultaneous MP/BI not less than 75 % and BPP not less than 0.9) at the nodes.

## Results

The nLSU datasets included 23 representatives genera of Hymenochaetaceae and the combined ITS+nLSU+RPB2 datasets included 41 fungal specimens representing 20 species. In addition to sequences of new species, 14 new sequences of three species without published DNA sequences were uploaded – *F. punctatiformis* (Murrill) Zmitr., Malysheva & Spirin, *F. setifer* (T. Hatt.) Y.C. Dai and *F. yunnanensis* Y.C. Dai.

The nLSU dataset had an aligned length of 1386 characters, of which 996 were constant, 96 were variable but parsimony-uninformative, and 294 were parsimony-informative. Maximum Parsimony (MP) analysis yielded four equally most parsimonious trees (TL = 1639, CI = 0.350, RI = 0.733, RC = 0.256, HI = 0.650). Bayesian (BI) resulted in a similar consensus tree to that of the Maximum Parsimony (MP) and Maximum Likelihood (ML) analysis, with 1 million generations and an average standard deviation of split frequencies = 0.009570.

The three-gene dataset had an aligned length of 2950 characters, of which 1990 were constant, 90 were variable but parsimony-uninformative, and 870 were parsimony-informative. Maximum Parsimony (MP) analysis yielded 4 most parsimonious trees with near-identical topologies (TL = 2631, CI = 0.569, RI = 0.807, RC = 0.459, HI = 0.431). Bayesian (BI) resulted in a similar consensus tree to that of the Maximum Parsimony (MP) and Maximum Likelihood (ML) analysis, with 1 million generations and an average standard deviation of split frequencies = 0.005640.

Eighteen species of *Fuscoporia* formed a well-supported clade (94/1/96 in Fig. 1) within the Hymenochaetaceae. *Fuscoporia* is a sister genus to *Coniferiporia*. Two samples from southern China are clustered into a new highly supported lineage (100/1/100 in Fig. 2) and in a clade with *F. ferrea* (Pers.) G. Cunn., *F. punctatiformis*, *F. subferrea* Q. Chen & Y. Yuan, and *F. yunnanensis* with high support (99/1/98 in Fig. 1; 100/1/100 in Fig. 2); it is described as *F. ramulicola* sp. nov. Another three specimens formed a distinct lineage with strong support (100/1/100 in Fig. 2) in *Fuscoporia*. This clade is interpreted as a new species, *F. Acutimarginata* sp. nov.

### Taxonomy

#### Fuscoporia acutimarginata

Taxon classificationFungiHymenochaetalesHymenochaetaceae

Y.C. Dai & Q. Chen sp. nov.

4298BC90-BB1A-58F6-84B0-C050C6D59056

824732

Figs 3A , 4

##### Type.

China. Yunnan Province: Kunming, Wild Duck Park, 2 August 2016, on fallen angiosperm branch, Dai 16892 (holotypes: BJFC 022998).

##### Etymology.

“*Acutimarginata*” (Latin): referring to the species with a sharp margin of fruiting body.

##### Description.

Basidiocarps annual, effused-reflexed to pileate, broadly attached, without taste or odor and soft corky when fresh. Pilei conchate, laterally fused, convex towards margin, projecting up to 1.5 cm, 7 cm wide and 6 mm thick at base. Pileal surface yellowish brown to dark brown, velutinate, concentrically sulcate with zoned; margin acute, yellowish brown. Pore surface yellowish brown when dry, glancing; margin distinct, yellowish, up to 2 mm wide; pores circular to angular, 5–7 per mm; dissepiments thin, entire. Context yellowish brown to dull brown, corky, up to 3 mm thick. Tubes yellowish brown, paler than context, corky, up to 3 mm long.

##### Hyphal structure.

Hyphal system dimitic; generative hyphae simple septate; tissue darkening but otherwise unchanged in KOH.

##### Subiculum.

Generative hyphae rare, hyaline to pale yellowish, thin- to slightly thick-walled, occasionally branched, 2–3.5 μm in diam; skeletal hyphae dominant, yellowish brown, thick-walled with a wide lumen, unbranched, aseptate, interwoven, 2–4.3 μm in diam.

##### Tubes.

Generative hyphae hyaline, thin-walled, occasionally branched, 2–3 μm in diam, occasionally encrusted at dissepiment edges; skeletal hyphae dominant, yellowish brown, thick-walled with a wide lumen, unbranched, aseptate, straight, subparallel along the tubes, 2–4 μm in diam. Irregular crystals occasionally present among trama and hymenia.

##### Hymenium.

Hymenial setae rare, mostly originating from tramal hyphae, subulate, dark brown, thick-walled, 20–40 × 3–7 μm; cystidioles frequent, fusoid, sometimes covered with crystals, hyaline, thin-walled, 16.5–26 × 4–6.5 μm; basidia broadly clavate, with four sterigmata and a simple septum at the base, 14–17 × 4.8–6.5 μm; basidioles similar in shape to basidia, but slightly smaller. Basidiospores cylindrical, hyaline, thin-walled, smooth, IKI–, CB–, (7–)7.5–9(–9.8) × (2.2–)2.5–3.2 μm, L = 8.12 μm, W = 2.87μm, Q = 2.73–2.95 (n = 60/2).

##### Additional specimens examined (paratypes).

China. Hunan Province: Yizhang County, Mangshan Nature Reserve, Guizizhai, 16 Aug 2014, on fallen angiosperm trunk, Dai 15115 (BJFC 018227), Dai 15137 (BJFC 018253).

**Figure 3.**
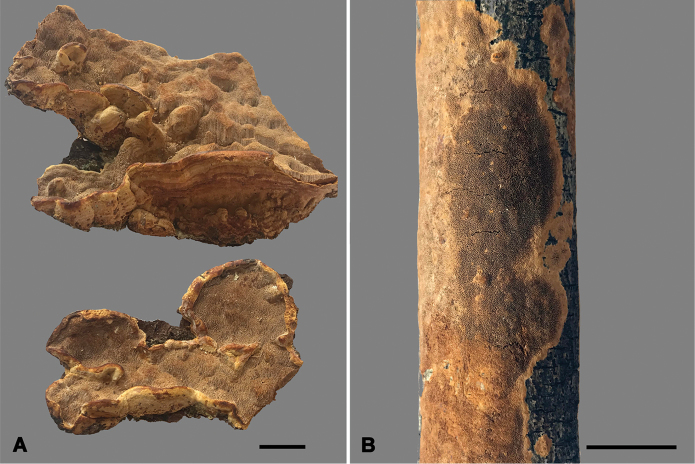
Basidiocarps of *Fuscoporia*. **A***F. acutimarginata* (Dai 15137) **B***F. ramulicola* (Dai 16155). Scale bars: 10 mm.

**Figure 4.**
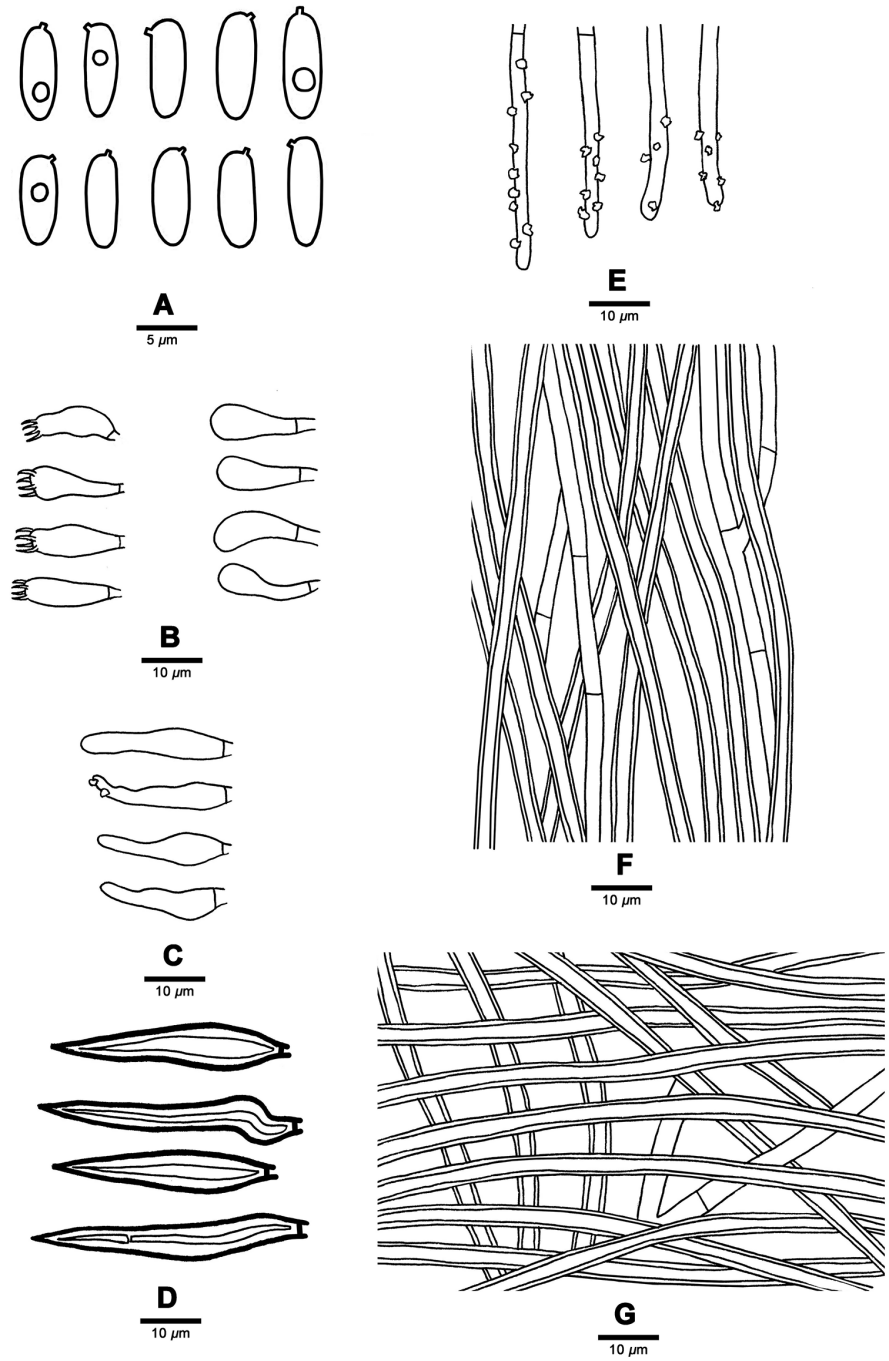
Microscopic structures of *Fuscoporia acutimarginata* (Holotype, Dai 16892) **A** basidiospores **B** basidia and basidioles **C** cystidioles **D** hymenial setae **E** generative hyphae at dissepiment edge **F** hyphae from trama **G** hyphae from subiculum.

#### Fuscoporia ramulicola

Taxon classificationFungiHymenochaetalesHymenochaetaceae

Y.C. Dai & Q. Chen sp. nov.

A45B5EAD-2319-5EAD-A1B3-1EA8E6AA1096

824734

Figs 3B , 5

##### Type.

China. Yunnan Province: Binchuan County, Jizushan Park, 30 August 2015, on fallen angiosperm branch, Dai 15723 (holotypes: BJFC 019827).

##### Etymology.

“*Ramulicola*” (Latin) referring to the species growing on branches.

##### Description.

Basidiocarps annual, resupinate, effused, inseparable, without taste or odor and corky when fresh, light-weight and hard corky when dry, up to 10 cm long, 2.2 cm wide and 1 mm thick at center. Pore surface grayish brown, fawn, cracked with age; sterile margin yellowish brown to olivaceous buff, distinctly paler than tubes, up to 1 mm wide; pores more or less angular, 6–7 per mm; dissepiments thin, sometimes irregular to slightly lacerate; abundant setae seen in tube cavities (under lens). Subiculum reddish brown, corky, very thin, about 0.1 mm thick. Tubes olivaceous buff, paler contrasting with pores and subiculum, hard corky, up to 0.9 mm long.

##### Hyphal structure.

Hyphal system dimitic; generative hyphae simple septate; tissue darkening but otherwise unchanged in KOH.

##### Subiculum.

Generative hyphae rare, hyaline, thin-walled, occasionally branched and simple septate, 2.5–3 μm in diam; skeletal hyphae dominant, rust-brown, thick-walled with a wide lumen, unbranched, aseptate, flexuous, loosely interwoven, 3–3.8 μm in diam.

##### Tubes.

Generative hyphae rare, mostly present at dissepiment edges and subhymenium, hyaline, thin-walled, occasionally branched and frequently simple septate, 1.8–2.8 μm in diam, some of them at dissepiment edges and in the hymenium encrusted with small crystals; skeletal hyphae dominant, yellowish brown, thick-walled with a wide lumen, unbranched, aseptate, flexuous to more or less straight, subparallel along the tubes, 2.5–3.8 μm in diam. Irregular crystals usually present among trama and hymenia.

##### Hymenium.

Hymenial setae frequent, mostly originating from hymenium, subulate, dark brown, thick-walled, 35–60 × 4.5–7 μm; cystidioles fusoid, sometimes covered with crystals, hyaline and thin-walled, 15–22 × 3–5 μm; basidia barrel-shaped, with four sterigmata and a simple septum at the base, 9–11 × 4.5–5.5 μm; basidioles frequently in hymenium, similar in shape to the basidia, but slightly smaller. Basidiospores cylindrical, hyaline, thin-walled, smooth, usually glued in tetrads, IKI–, CB–, with some small guttules, (5.2–)5.8–7(–7.2) × (1.8–)2–2.5(–2.8) μm, L = 6.37 μm, W = 2.28 μm, Q = 2.57–2.88 (n = 60/2).

##### Additional specimen examined (paratype).

China. Hainan Province: Wuzhishan County, Wuzhishan Nature Reserve, 14 Nov 2015, on fallen angiosperm branch, Dai 16155 (BJFC 020252).

**Figure 5.**
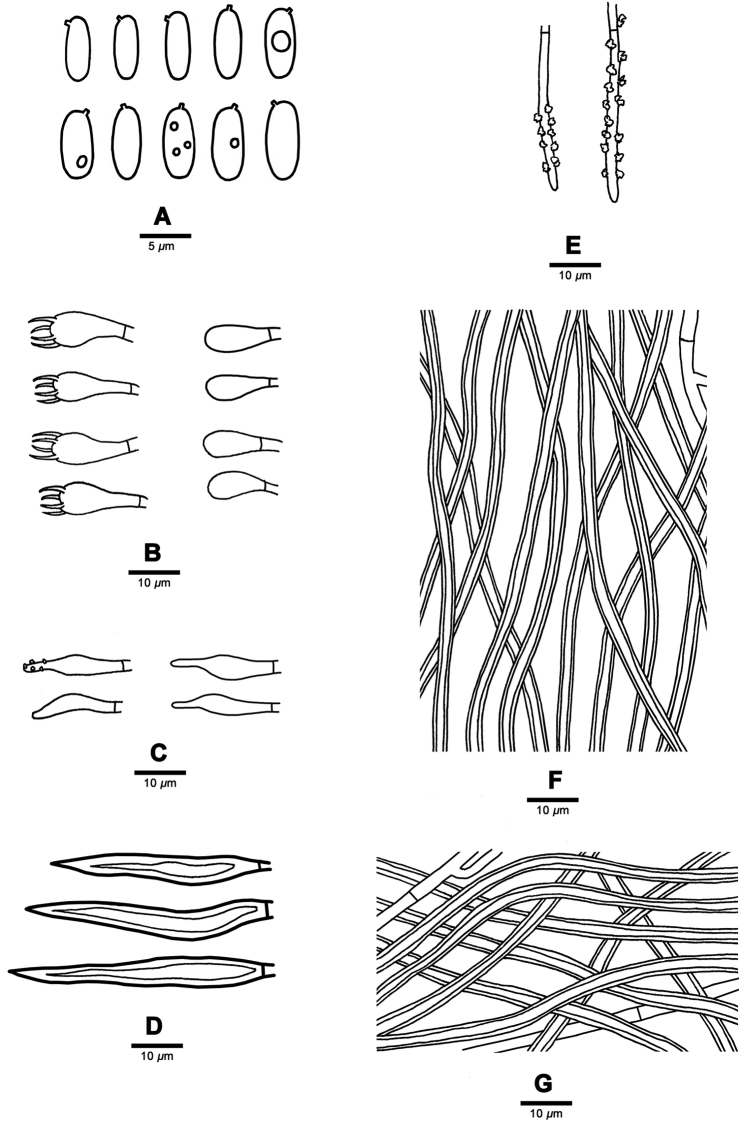
Microscopic structures of *Fuscoporia ramulicola* (Holotype, Dai 15723) **A** basidiospores **B** basidia and basidioles **C** cystidioles **D** hymenial setae **E** generative hyphae at dissepiment edge **F** hyphae from trama **G** hyphae from subiculum.

## Discussion

In the study, sixteen previously accepted species of *Fuscoporia* were referred to morphological examination and phylogenetic analyses. Two species of *Fuscoporia* from China, *F. acutimarginata* and *F. ramulicola*, are described as new on the basis of molecular data and morphology. *F. acutimarginata* is characterized by annual, effused-reflexed to pileate basidiocarps, small pores (5–7 per mm), a dimitic hyphal structure, hymenial setae rarely present, originating from tramal hyphae, the presence of cystidioles, and cylindrical basidiospores measuring 7.5–9 × 2.5–3.2 μm. Phylogenetically, samples of *F. acutimarginata* formed a well-supported monophyletic lineage distinct from other *Fuscoporia* species (Fig. 2). *F. acutimarginata* is very similar to *F. setifer* in having annual, effused-reflexed basidiocarps and the presence of cystidioles, but the latter has bigger pores (3–4 per mm) and smaller basidiospores (5.8–7 × 2–2.5 μm, Dai 2010). *F. acutimarginata* has a similar macromorphology to *F. gilva* (Schwein.) T. Wagner & M. Fisch., but the latter has frequently septate skeletal hyphae and ellipsoid to ovoid basidiospores (4–5 × 3–3.5 μm, Ryvarden 2004).

*Fuscoporia ramulicola* is distributed in southern China and characterized by annual and resupinate basidiocarps, small pores (6–7 per mm), a dimitic hyphal system, subulate hymenial setae, the presence of cystidioles, and cylindrical basidiospores measuring 5.8–7 × 2–2.5 μm. *F. ferrea*, *F. subferrea*, *F. yunnanensis* and *F. ramulicola* have overlapping distribution in China and clustered together with *F. punctatiformis* in a clade with strong support (99/1/98 in Fig. 1, 100/1/100 in Fig. 2). They are distinguishable according to their morphology and molecular data: *F. ferrea* is distinguished from *F. ramulicola* by its perennial basidiocarps and widely distributed in northern China, Europe and North America (Ryvarden and Gilbertson 1994, Lowe 1966, Chen et al. 2019); *F. subferrea* has smaller pores (7–10 per mm) and shorter basidiospores (4.2–6.2 × 2–2.6 μm, Chen and Yuan 2017); *F. yunnanensis* has larger pores (2–4 per mm) and wider basidiospores (6–8.3 × 2.4–3 μm, Dai 2010); *F. punctatiformis* has shorter hymenial setae (18–25 μm vs. 35–60 μm), subcylindrical basidiospores with a pointed apex (4–6 × 1.5–2 μm, Ryvarden and Johansen 1980) and is reported from America, Brazil and USA (Ryvarden 2004; Groposo et al. 2007). *Fuscoporia ramulicola* is similar to *F. contigua* and *F. ferruginosa* in having resupinate basidiocarps; however, *F. contigua* and *F. ferruginosa* have mycelial, extra-hymenial setae (Chen et al. 2019); in addition, the three species are distantly related in our phylogenies (Figs 1, 2). *Fuscoporia montana* Y.C. Dai & Niemelä and *F. chrysea* (Lév.) Baltazar & Gibertoni are also similar to *F. ramulicola* in sharing resupinate basidiocarps, abundant subulate hymenial setae, absence of mycelial setae and distributed in southern China. *Fuscoporia montana* differs from *F. ramulicola* in having ovoid basidiospores 6.5–8.2 × 3.2–4.2 μm (Niemelä et al. 2001) whereas *F. chrysea* is different from *F. ramulicola* by its smaller pores (9–10 per mm) and shorter basidiospores (3–4 × 2–2.5 μm, Dai 2010).

### Key to *Fuscoporia* species in China

**Table:** 

1	Basidiocarps usually laterally stipitate; hymenial setae absent	***F. discipes***
–	Basidiocarps sessile; hymenial setae present	**2**
2	Basidiocarps completely resupinate	**3**
–	Basidiocarps effuse-reflexed to pileate	**11**
3	Mycelial setae present in the decayed wood and margin of basidiocarps (by hand lens)	**4**
–	Mycelial setae absent from the decayed wood and margin of basidiocarps (by hand lens)	**5**
4	Pores 7–8 per mm	***F. ferruginosa***
–	Pores 2–3 per mm	***F. contigua***
5	Basidiocarps perennial	**6**
–	Basidiocarps annual	**9**
6	Pores 5–7 per mm	**7**
–	Pores 7–10 per mm	**8**
7	Basidiospores cylindrical, 6–7.8 × 2–2.5 μm	***F. ferrea***
–	Basidiospores subcylindrical, 4–6 × 1.5–2 μm	***F. punctatiformis***
8	Pores 9–10 per mm; basidiospores ellipsoid, < 5 μm long	***F. chrysea***
–	Pores 7–8 per mm; basidiospores narrowly ovoid, > 5 μm long	***F. montana***
9	Pores 2–4 per mm	***F. yunnanensis***
–	Pores 6–10 per mm	**10**
10	Pores 7–10 per mm; basidiospores 4.2–6.2 µm long	***F. subferrea***
–	Pores 6–7 per mm; basidiospores 6–7 µm long	***F. ramulicola***
11	Hymenial setae usually hooked	***F. wahlbergii***
–	Hymenial setae straight	**12**
12	Pores 3–4 per mm	***F. setifera***
–	Pores 5–11 per mm	**13**
13	Basidiocarps annual, margin acute	**14**
–	Basidiocarps perennial, margin obtuse	**15**
14	Skeletal hyphae septate, spores ellipsoid, 3.3–4.2 × 2.2–3 μm	***F. gilva***
–	Skeletal hyphae aseptate, spores cylindrical, 7.5–9 × 2.5–3.2 μm	***F. acutimarginata***
15	Basidiocarps subungulate; contextual skeletal hyphae aseptate	***F. torulosa***
–	Basidiocarps usually applanate; contextual skeletal hyphae septate	**16**
16	Basidiospores 3.3–4.1 × 2.1–2.4 μm, skeletal hyphae unchanged in KOH	***F. rhabarbarina***
–	Basidiospores 4–4.8 × 3.6–3.9 μm, skeletal hyphae swelling in KOH	***F. senex***

## Supplementary Material

XML Treatment for Fuscoporia acutimarginata

XML Treatment for Fuscoporia ramulicola
